# The anatomy of COVID-19 comorbidity networks among hospitalized Korean patients

**DOI:** 10.4178/epih.e2021035

**Published:** 2021-05-07

**Authors:** Eun Kyong Shin, Hyo Young Choi, Neil Hayes

**Affiliations:** 1 Department of Sociology, Korea University, Seoul, Korea; 2 Department of Preventive Medicine, University of Tennessee Health Science Center, Memphis, TN, USA; 3 Department of Medicine, Division of Hematology and Oncology, University of Tennessee Health Science Center, Memphis, TN, USA

**Keywords:** COVID-19, Korea, Comorbidity, Social network analysis, Infectious disease, Symptoms

## Abstract

**OBJECTIVES:**

We aimed to examine how comorbidities were associated with outcomes (illness severity or death) among hospitalized patients with coronavirus disease 2019 (COVID-19).

**METHODS:**

Data were provided by the National Medical Center of the Korea Disease Control and Prevention Agency. These data included the clinical and epidemiological information of all patients hospitalized with COVID-19 who were discharged on or before April 30, 2020 in Korea. We conducted comorbidity network and multinomial logistic regression analyses to identify risk factors associated with COVID-19 disease severity and mortality. The outcome variable was the clinical severity score (CSS), categorized as mild (oxygen treatment not needed), severe (oxygen treatment needed), or death.

**RESULTS:**

In total, 5,771 patients were included. In the fully adjusted model, chronic kidney disease (CKD) (odds ratio [OR], 2.58; 95% confidence interval [CI], 1.19 to 5.61) and chronic obstructive pulmonary disease (COPD) (OR, 3.19; 95% CI, 1.35 to 7.52) were significantly associated with disease severity. CKD (OR, 5.35; 95% CI, 2.00 to 14.31), heart failure (HF) (OR, 3.15; 95% CI, 1.22 to 8.15), malignancy (OR, 3.38; 95% CI, 1.59 to 7.17), dementia (OR, 2.62; 95% CI, 1.45 to 4.72), and diabetes mellitus (OR, 2.26; 95% CI, 1.46 to 3.49) were associated with an increased risk of death. Asthma and hypertension showed statistically insignificant associations with an increased risk of death.

**CONCLUSIONS:**

Underlying diseases contribute differently to the severity of COVID-19. To efficiently allocate limited medical resources, underlying comorbidities should be closely monitored, particularly CKD, COPD, and HF.

## INTRODUCTION

With the growing spread of coronavirus disease 2019 (COVID-19), the complex and varying fatality rates call for a new perspective on pandemics [1-3]. The alarming rate of growth of the COVID-19 pandemic has highlighted a lack of understanding of factors that influence disease severity and mortality. The global diffusion paths and regionally distinctive transmission patterns highlight the complex nature of this 21st-century epidemic [4]. Severe acute respiratory syndrome coronavirus 2 (SARS-CoV-2), the virus that causes COVID-19, is not only novel but also displays large variations in mortality rates across regions [2,5-8]. Case fatality varies greatly both within and among countries. This heterogeneity in the fatality rate may be explained by differences in population demographics [9,10], access to adequate medical care [10,11], and the presence of comorbidities. The prevalence of comorbidities in COVID-19 cases varies widely across countries and populations [7,12-15]. In-depth understanding of the importance of underlying disease is urgently needed to efficiently identify the patients at highest risk and thus improve medical resource allocation and distribution.

In particular, understanding networks of underlying diseases can help identify at-risk populations. Rather than focusing on the prevalence of a comorbidity in a single segment of the population, comorbidity networks can be used to depict relative centralities and efficiently pinpoint the risk [16,17]. Past studies have shown that comorbidities are significantly associated with clinical outcomes of COVID-19 [5,7,18-22]. The most common comorbidities among confirmed patients are diabetes mellitus (DM) [23,24] and hypertension (HTN) [25], while chronic obstructive pulmonary disease (COPD) and cancer are also associated with higher mortality and poor outcomes [19]. Cancer patients are also thought to be particularly vulnerable to infection [26]. The high prevalence of HTN among patients with COVID-19 is not surprising; HTN is common in older people, who appear to have an elevated risk of experiencing severe disease and its complications [27-29]. However, the relevance of pre-existing comorbidities to fatality risk requires further understanding. We investigated the comorbidities and symptom networks of hospitalized COVID-19 patients and the associations with clinical severity and mortality from COVID-19.

## MATERIALS AND METHODS

### Data source

The Korea Disease Control and Prevention Agency provided the data for this study. These data included basic information, laboratory results at the time of initial examination, the hospitalization history, hospital conditions, clinical severity, general blood test results, and history of chronic disease.

### Study patients and covariates

We included all patients who were confirmed to have COVID-19, hospitalized, and discharged in Korea between January 21, 2020 and April 30, 2020, excluding 14 cases involving pregnancy, 27 cases with no clinical severity reported, and 4 cases with incomplete data. The final sample included 5,571 patients.

### Exposure of interest

This study was focused on the presence of comorbidities. We included the following common comorbid conditions reported by patients at the time of hospitalization: DM, HTN, heart failure (HF), cardiac conduction disease (CCD), asthma, COPD, chronic kidney disease (CKD), malignancy, chronic liver disease (CLD), rheumatic disease/autoimmune disorder (RDAD), and dementia. These underlying diseases are the most commonly observed comorbidities among COVID-19 patients [19,22].

### Outcomes

The outcome variable was the clinical severity score (CSS). The CSS was initially classified into 8 categories: 1 (no activity limitation), 2 (limited activity but no oxygen [O_2_] required), 3 (O_2_ with nasal prongs), 4 (O_2_ with facial mask), 5 (non-invasive ventilation), 6 (invasive ventilation), 7 (multiorgan failure/extracorporeal membrane oxygenation), and 8 (death). Few cases (n = 106, 1.9%) were categorized between 4 and 7 (4, n= 43; 5, n= 33; 6, n= 19; 7, n= 11), making statistical analyses using all the 8 levels of the variable invalid. To increase the statistical power with a balanced sample distribution, we converted this raw scale into new CSS categories. Risk factor analyses commonly involve dichotomous variables such as alive/dead or non-severe/severe. However, this could result in a loss of information regarding the original variable. To accurately examine the impacts of comorbidities on severity, we converted the raw scale into 3 new categories: 1, mild disease (O_2_ treatment not needed, original scale= 1-2); 2, severe disease (O_2_ treatment needed, original scale= 3-7); and 3, death (raw scale= 8).

### Statistical analysis

First, patients’ demographic characteristics were summarized in relation to the means and interquartile ranges of the CSS groups. We performed statistical tests to identify any significant inter-group differences with respect to symptoms and comorbidities.

Second, the relationships between COVID-19 severity and comorbidities were examined using 2-mode network analysis. Comorbidity prevalence was also summarized via a similar network analysis [30,31]. A single comorbidity was expected to yield a different CSS from 2 or more concurrent comorbidities. In the network, nodes represent other diseases present among the COVID-19 patients. Lines (links) indicate the coappearance of 2 or more underlying diseases in a patient. A comorbidity network, which is essentially a constellation of diseases, can effectively indicate the relative prevalence rates of underlying diseases [17]. The comorbidity networks for severe disease and death were compared with those for mild disease. Since the COVID-19 disease course unfolds differently according to the pathological history of each individual, simply understanding the virus is not enough to understand individual variation in response to the virus. Applying a network perspective helps elucidate the complex pathodynamics of COVID-19. A symptom or chronic disease network should assist healthcare workers in more easily identifying groups at highrisk for poor outcomes of COVID-19. We compared the relative connectivity of each node with other underlying diseases using eigenvector and weighted degree centralities. Eigenvector centrality is a measure of the influence of a node in a network. Relative centrality scores are assigned to each node based on its connectivity to other nodes. Weighted degree centrality refers to the sum of the weights assigned to capture the strengths of the links to other directly connected diseases. While eigenvector centrality can be used to identify how diseases are commonly connected to all other diseases, weighted degree centrality can capture the strength of these connections. We used both measures to depict the COVID-19 comorbidity network structure.

Next, we used the correlation structure between each pair of comorbidity and symptom variables to identify any associations. A p-value was derived for each of the estimated correlation coefficients based on the Pearson correlation test [32]. The statistical significance of the association was described using edge bundling for graph visualization. This analysis was used to investigate the association between each underlying disease and the risk of developing COVID-19 symptoms.

Finally, we conducted multinomial logistic regression analysis [33-35] to identify risk factors associated with illness severity and mortality among COVID-19 patients by comparing the severe illness and death CSS groups with the mild group. This enabled an in-depth examination that may have been obscured if we had used only 2 categories, such as survival and death. After all, some comorbidities may be associated with severe disease but not lead to death, or vice versa. Our outcomes of interest were the risk factors statistically associated with the presence of severe shortness of breath (SOB); therefore, ventilation treatment was included as a risk factor, as were the risk factors associated with death. Two levels of model adjustment were used: age, sex, and body mass index (BMI) (the most common control variables in the existing comorbidity studies [7,22,36]) only (model 1) and additional adjustment of model 1 for all of the comorbidity covariates (model 2). All statistical analyses were performed using R version 4.0.0 (R Foundation for Statistical Computing, Vienna, Austria), and graphical visualization was performed with Gephi software. The multinomial function from the nnet package was used to construct multinomial logistic regression models. We considered a pvalue < 0.05 to indicate statistical significance.

### Ethics statement

This study was approved by the Korea University Institutional Review Board (IRB-2020-0177).

## RESULTS

A total of 5,571 patients were included in this study (Tables 1 and 2), of whom 3,267 (58.6%) were female and 2,304 (41.4%) were male. A total of 1,770 patients (31.8%) were over 60 years old. HTN was the most common comorbid condition (21.5%), followed by DM (12.3%), dementia (4.0%), and CCD (3.2%). Other comorbidities included malignancy (2.6%), asthma (2.3%), CLD (1.5%), HF (1.0%), CKD (1.0%), COPD (0.7%), and RDAD (0.7%). Cough (41.7%) was the most common symptom, followed by sputum production (28.8%), fever (23.3%), and headache (17.3%). Other symptoms included muscle aches (16.4%), sore throat (15.6%), SOB (11.9%), rhinorrhea (11.0%), diarrhea (9.2%), vomiting and nausea (VN; 4.4%), fatigue (4.2%), and alteration of consciousness (ACC; 0.6%).

We converted the raw COVID-19 severity scale (CSS) scores into 3 new categories: 1, mild disease (O_2_ treatment not needed, original scale= 1-2); 2, severe disease (O_2_ treatment needed, original scale= 3-7); and 3, death (raw scale= 8). Based on this new scale, 4,762 patients (85.5%) did not require O_2_ treatment (CSS= 1), 575 patients (10.3%) required O_2_ treatment (CSS = 2), and 234 patients (4.2%) died (CSS= 3). Using the unadjusted multinomial logistic models, age, sex, BMI, every symptom, and every comorbidity except CLD were found to be associated with CSS. Consistent with previous studies, older patients and male patients had significantly higher CSS scores than younger and female patients, respectively. COVID-19 patients with pre-existing DM, HTN, HF, asthma, COPD, CKD, RDAD, or dementia were more likely to be in a critical condition requiring ventilation and had higher mortality. Patients with CCD did not have a significantly increased risk of SOB, but did have higher mortality. In contrast, patients with malignancy were more likely to experience increased SOB, but they were not more likely to die.

### Comorbidities and COVID-19 symptoms

**Figure 1.**
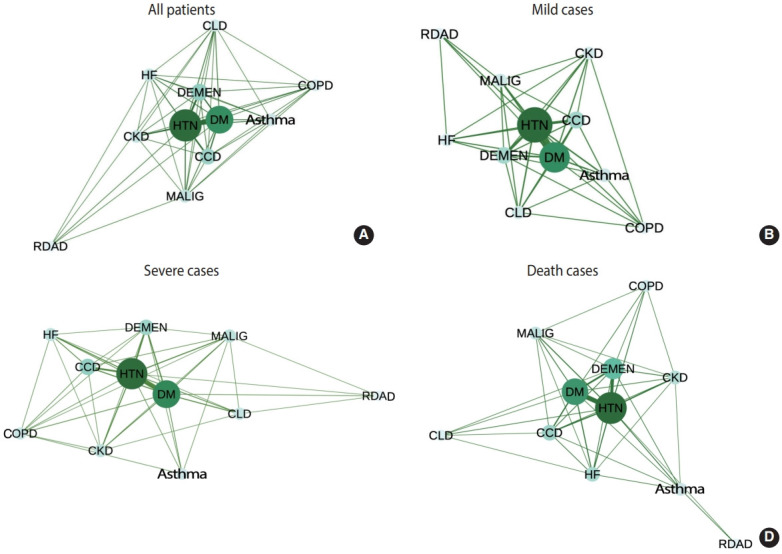
Coronavirus disease 2019 (COVID-19) comorbidity network by severity (A) all patients, (B) mild cases, (C) severe cases, and (D) death cases. Nodes are major comorbidities and lines represent simultaneous presence of two conditions in a patient. The size of nodes represents the weighted degree centralities of the co-observed chronic conditions, and the thickness of the lines represent the prevalence of the co-exhibition of the two conditions. DM, diabetes mellitus; HTN, hypertension; HF, heart failure; CCD, cardiac conduction disease; COPD, chronic obstructive pulmonary disease; CKD, chronic kidney disease; MALIG, malignancy; CLD, chronic liver disease; RDAD, rheumatic disease/autoimmune disorder; DEMEN, dementia.

Figure 1 presents the comorbidity networks for the hospitalized COVID-19 patients. HTN and DM were the most common underlying diseases. The overall comorbidity network is shown in Figure 1A. These comorbidities were not only most common among COVID-19 patients, but they also often appeared concomitantly with other chronic diseases. The comorbidity networks for mild disease (n= 4,762), severe disease (n= 575), and death (n= 234) are shown in Figure 1B-1D. As severity increased, centralities of disease severity emerged more prominently as key nodes in the network. The main components of the severe and fatal networks were HTN, DM, CCD, and dementia. Thus, the severe and fatal cases had distinct underlying comorbidity patterns compared to the mild cases. The eigenvector centrality and weighted degree centrality of each comorbidity are summarized in Table 3. Very little variation in degree centrality was present among the 11 underlying diseases because they were commonly co-observed among the confirmed COVID-19 patients. The clinical importance of a disease is indicated by a significant difference in its centrality across severity groups. Although high prevalence alone does not mean that a disease is a critical contributor to increased disease severity, its relative connectivity to other underlying diseases is an important factor for identifying patients at greater risk. For example, although HTN was the most common disease present among the hospitalized COVID-19 patients, the relative centrality of HTN was particularly highly pronounced among the fatal cases. HF was also found to be one of the most prevalent comorbidities, yet its relative centrality among patients with mild and severe cases was low. DM was the most central underlying disease among the mild and severe cases.

**Figure 2.**
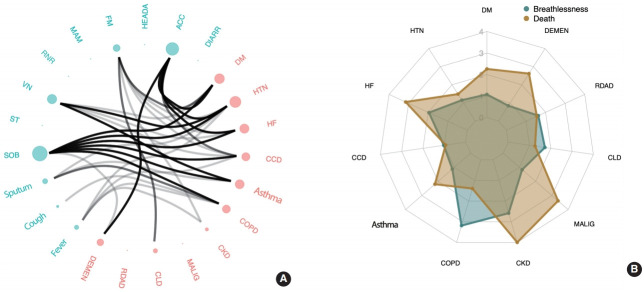
Association between comorbidities and clinical manifestations of coronavirus disease 2019 (COVID-19) patients. (A) Statistical significance for the correlation between each pair of the comorbidities (pink) and COVID-19 symptoms (sky blue) was presented using an edge bundling graph. The density of the lines represents the strength of the statistical significance with a dense line indicating stronger association (a smaller p-value). (B) The radar chart exhibits the odds ratios estimated by the multinomial logistic regression model used in Materials Methods. The blue radar shows the results with respect to severe cases and the brown radar shows the results for death cases. Overall, the brown radar encloses the blue one, suggesting that the impacts of the comorbidities are generally higher for the death cases compared to the cases with severe breath difficulties. Some different shape patterns were observed with the highest discrepancy for COPD, which indicates that COPD is strongly associated with severe breathless but not with death. SOB, shortness of breath; ST, sore throat; VN, vomiting and nausea; RNR, rhinorrhea; MAM, muscle ache; FM, fatigue; HEADA, headache; ACC, alteration of consciousness; DIARR, diarrhea; DM, diabetes mellitus; HTN, hypertension; HF, heart failure; CCD, cardiac conduction disease; COPD, chronic obstructive pulmonary disease; CKD, chronic kidney disease; MALIG, malignancy; CLD, chronic liver disease; RDAD, rheumatic disease/autoimmune disorder; DEMEN, dementia.

Figure 2A shows the associations between comorbidities and COVID-19 symptoms. The comorbidity most commonly and strongly associated with multiple symptoms was HTN, followed by DM, HF, asthma, and CCD. Among the reported symptoms, SOB consistently showed a strong correlation with comorbidities, including DM, HTN, HF, CCD, asthma, and COPD. Although SOB was exhibited in only 11.9% of the reported cases in our study (a relatively small proportion compared to cough, fever, and sputum), it is noteworthy that it was the most prevalent symptom strongly associated with comorbidities.

Regarding the impact of comorbidities on symptoms, COVID-19 patients with HTN were at a greater risk of having 1 or more of these symptoms: ACC, VN, SOB, fatigue, sputum production, and fever, listed in the order of the strongest to the weakest association (p < 0.05). Patients with DM were more likely to have ACC, SOB, fatigue, and VN than patients without DM. HF was associated with ACC, SOB, and VN. Patients with asthma showed an increased risk of experiencing SOB, VN, and sputum production. Patients with CCD were more likely to experience SOB, ACC, VN, and fever. COPD was associated with a higher risk of SOB, sputum production, cough, and fever. Patients with CKD were more likely to experience fatigue and fever. CLD was only associated with fatigue, and dementia was associated with ACC and SOB.

### Multinomial logistic regression (severe disease and death)

In model 1 (adjusted for age, sex, and BMI only), CKD and HF were significant risk factors for both severe disease and mortality. DM, HTN, malignancy, and dementia showed strong associations with increased mortality alone, whereas COPD was associated with severe disease alone.

Model 2 included additional adjustment for all of the comorbidity covariates (Table 4 and Figure 2B). In this model, age and sex (female) were associated with both severe disease and death, as was CKD, as is consistent with the results from model 1. Patients with COPD had a greater risk of developing breathing difficulties, but no significantly higher risk of death. DM, HF, malignancy, and dementia were strongly associated with the risk of death.

More specifically, the risk of severe disease increased with the presence of COPD (odds ratio [OR], 3.19; 95% confidence interval [CI], 1.35 to 7.52) and CKD (OR, 2.58; 95% CI, 1.19 to 5.61). HF, which was associated with severe breathing difficulties in model 1, showed a weaker association in model 2. The risk of death was most strongly associated with CKD (OR, 5.35; 95% CI, 2.00 to 14.31), followed by malignancy (OR, 3.38; 95% CI, 1.59 to 7.17), HF (OR, 3.15; 95% CI, 1.22 to 8.15), dementia (OR, 2.62; 95% CI, 1.45 to 4.72), and DM (OR, 2.26; 95% CI, 1.46 to 3.49). Other comorbidities rather weakly associated (p< 0.1) with increased mortality were asthma and HTN, although the associations were not statistically significant at a level of significance of 0.05 (asthma: OR, 2.20; 95% CI, 0.86 to 5.59; p= 0.10 and HTN: OR, 1.49; 95% CI, 0.95 to 2.31; p= 0.08).

## DISCUSSION

This study was a national case series focused on the complexity of comorbidities among hospitalized COVID-19 patients. Relative to the general population, the included patients were relatively likely to be older, to be female, and to have pre-existing HTN and/or DM. However, the presence of underlying disease was not always associated with increased disease severity. Although HTN was the most common comorbidity in this study, it was not a statistically significant factor in explaining severity after controlling for other comorbidities. Thus, prevalence alone does not explain symptom severity.

While everyone may be equally susceptible to SARS-CoV-2, symptom progression after infection is not homogeneous. Identifying the comorbidities that are more likely to cause deterioration in a patient’s condition can be vital for the efficient use of healthcare resources. Most hospitalized patients in this study had at least 1 comorbidity. An increase in the presence of comorbidities may explain the wide variation in COVID-19 mortality across regions and age groups. Therefore, even among people exposed to the same virus, the clinical outcomes may vary.

This study had several limitations. First, the data were collected from an electronic health record database, which fails to include more detailed information that may be present in manual medical records. The comorbidity variables were dummy variables in our study. Second, we studied only hospitalized patients who had been discharged, potentially excluding severely ill patients who were admitted for extended periods. The total number of confirmed COVID-19 patients in Korea by April 30, 2020 was 10,765, with 247 deaths. Therefore, our study included only 51.7% of all confirmed cases and 94.7% of all deaths. The prevalence of many comorbidities is known to increase with age. In this study, age was categorized in 10-year bands, which could be too wide of an interval to capture more subtle within-band differences. Therefore, age might not have been completely adjusted for, causing our model to overestimate the association between the presence of comorbidities and disease severity. Lastly, this study did not include information regarding residential area. Although the overall comorbidity patterns between patients from Daegu and patients from other areas have been found to be consistent [22], chronic affinity may exhibit different geo-prevalence [37]. More research with finer geo-granularity is needed to unpack the influence of residential area on the epidemic and patients’ clinical course.

In this study, we explored the complexities of COVID-19 comorbidities to understand their contributions to disease severity. We found that the high prevalence of a comorbidity did not necessarily contribute to COVID-19 severity. However, the presence of underlying comorbidities had a varied effect on disease severity in COVID-19 patients. Insight into underlying diseases that critically contribute to COVID-19 severity is important in designing an efficient pandemic control policy. In the present study, HTN and DM appeared to be dominant underlying factors, and both are preventable chronic diseases. Healthcare systems should provide programs for the early detection and effective treatment of these conditions and maximize public educational opportunities. Routine management of chronic diseases could serve as an important social immunity mechanism, even in the unprecedented event of an infectious disease crisis.

## Figures and Tables

**Table 1.** Baseline characteristics for CSS, age, sex, and BMI, mean and interquartile range of CSS, non-parametric rank test

Characteristics	n (%)	CSS, mean [Q1, Q3]	p-value
CSS			
1 (mild)	4,762 (85.5)	-	
2 (severe)	575 (10.3)	-	
3 (death)	234 (4.2)	-	
Age (yr)			
≤49	2,662 (47.8)	1.13 [1.00, 1.00]	<0.01
50-69	2,044 (36.7)	1.53 [1.00, 1.00]	
70-79	542 (9.7)	2.59 [1.00, 3.00]	
≥80	323 (5.8)	4.18 [1.50, 8.00]	
Sex			
Female	3,267 (58.6)	1.52 [1.00, 1.00]	0.05
Male	2,304 (41.4)	1.70 [1.00, 1.00]	
BMI (kg/m^2^)^1^			
<25	3,136 (71.5)	1.46 [1.00, 1.00]	<0.01
≥25	1,248 (28.5)	1.62 [1.00, 1.00]	

CSS, clinical severity score; BMI, body mass index.

1 Indicates variables with unavailable data points: BMI (1,187 not available).

**Table 2.** Baseline characteristics for symptoms and comorbidity, mean and interquartile range of CSS, non-parametric rank test

Characteristics	Present		Absent		p-value
	n (%)	CSS, mean [Q1, Q3]	n (%)	CSS, mean [Q1, Q3]	
Symptom					
Fever	1,297 (23.3)	2.03 [1.00, 3.00]	4,274 (76.7)	1.47 [1.00, 1.00]	<0.01
Cough	2,324 (41.7)	1.60 [1.00, 1.00]	3,247 (58.3)	1.60 [1.00, 1.00]	0.10
Sputum	1,604 (28.8)	1.67 [1.00, 1.00]	3,967 (71.2)	1.57 [1.00, 1.00]	<0.01
ST	869 (15.6)	1.33 [1.00, 1.00]	4,702 (84.4)	1.65 [1.00, 1.00]	<0.01
RNR	614 (11.0)	1.29 [1.00, 1.00]	4,957 (89.0)	1.64 [1.00, 1.00]	<0.01
MAM	915 (16.4)	1.51 [1.00, 1.00]	4,656 (83.6)	1.61 [1.00, 1.00]	0.87
FM	233 (4.2)	1.97 [1.00, 3.00]	5,338 (95.8)	1.58 [1.00, 1.00]	<0.01
HEADA	961 (17.3)	1.38 [1.00, 1.00]	4,610 (82.7)	1.64 [1.00, 1.00]	<0.01
ACC	32 (0.6)	6.06 [4.25, 8.00]	5,539 (99.4)	1.57 [1.00, 1.00]	<0.01
VN	243 (4.4)	1.95 [1.00, 3.00]	5,328 (95.6)	1.58 [1.00, 1.00]	<0.01
DIARR	515 (9.2)	1.59 [1.00, 1.00]	5,056 (90.8)	1.60 [1.00, 1.00]	0.87
SOB	662 (11.9)	3.03 [1.00, 4.00]	4,909 (88.1)	1.40 [1.00, 1.00]	<0.01
Comorbidity					
DM	686 (12.3)	2.50 [1.00, 3.00]	4,885 (87.7)	1.47 [1.00, 1.00]	<0.01
HTN	1196 (21.5)	2.39 [1.00, 3.00]	4,375 (78.5)	1.38 [1.00, 1.00]	<0.01
HF	58 (1.0)	3.88 [1.00, 8.00]	5,513 (99.0)	1.57 [1.00, 1.00]	<0.01
CCD^1^	179 (3.2)	2.61 [1.00, 3.00]	5,376 (96.5)	1.56 [1.00, 1.00]	<0.01
Asthma	128 (2.3)	2.05 [1.00, 2.00]	5,443 (97.7)	1.59 [1.00, 1.00]	<0.01
COPD	40 (0.7)	3.20 [1.00, 3.50]	5,531 (99.3)	1.59 [1.00, 1.00]	<0.01
CKD	55 (1.0)	3.84 [1.00, 8.00]	5,516 (99.0)	1.58 [1.00, 1.00]	<0.01
MALIG^1^	143 (2.6)	2.36 [1.00, 3.00]	5,427 (97.4)	1.58 [1.00, 1.00]	<0.01
CLD^1^	82 (1.5)	1.99 [1.00, 2.75]	5,166 (92.7)	1.62 [1.00, 1.00]	<0.01
RDAD^1^	38 (0.7)	1.92 [1.00, 1.75]	5,204 (93.4)	1.63 [1.00, 1.00]	0.37
DEMEN^1^	224 (4.0)	3.99 [2.00, 8.00]	5,021 (90.1)	1.52 [1.00, 1.00]	<0.01

CSS, clinical severity score; ST, sore throat; RNR, rhinorrhea; MAM, muscle ache; FM, fatigue; HEADA, headache; ACC, alteration of consciousness; VN, vomiting and nausea; DIARR, diarrhea; SOB, shortness of breath; DM, diabetes mellitus; HTN, hypertension; HF, heart failure; CCD, cardiac conduction disease; COPD, chronic obstructive pulmonary disease; CKD, chronic kidney disease; MALIG, malignancy; CLD, chronic liver disease; RDAD, rheumatic disease/autoimmune disorder; DEMEN, dementia; NA, not available.

1 Indicates variables with unavailable data points: CCD (16 NA), MALIG (1 NA), CLD (323 NA), RDAD (329 NA), DEMEN (326 NA).

**Table 3.** Centralities in comorbidity networks

Comorbidity	Eigenvector centralities			Weighted degree centralities		
	Mild illness (n=4,762)	Severe illness (n=575)	Death (n=234)	Mild illness (n=4762)	Severe illness (n=575)	Death (n=234)
DM	1.000	1.000	0.974	358	146	150
HTN	1.000	1.000	1.000	447	174	198
HF	0.662	0.777	0.974	28	27	41
CCD	0.868	0.807	0.893	108	54	51
Asthma	0.692	0.596	0.757	48	14	26
COPD	0.681	0.875	0.718	18	20	16
CKD	0.777	0.880	0.905	31	27	34
MALIG	0.764	0.912	0.819	44	28	26
CLD	0.780	0.643	0.613	40	22	10
RDAD	0.450	0.455	0.224	14	6	5
DEMEN	0.948	0.875	0.974	104	46	101

DM, diabetes mellitus; HTN, hypertension; HF, heart failure; CCD, cardiac conduction disease; COPD, chronic obstructive pulmonary disease; CKD, chronic kidney disease; MALIG, malignancy; CLD, chronic liver disease; RDAD, rheumatic disease/autoimmune disorder; DEMEN, dementia.

**Table 4.** Multinomial logistic regression for coronavirus disease 2019 (COVID-19) severity^1^

Characteristics	Model 1				Model 2			
	Severe illness	p-value	Death	p-value	Severe illness	p-value	Death	p-value
Age	-	-	-	-	1.80 (1.66, 1.95)	<0.01	3.44 (2.74, 4.31)	<0.01
Sex	-	-	-	-	1.39 (1.11, 1.72)	<0.01	3.06 (1.99, 4.72)	<0.01
BMI	-	-	-	-	1.27 (1.14, 1.42)	<0.01	1.13 (0.91, 1.41)	0.27
DM	1.19 (0.91, 1.55)	0.20	2.60 (1.73, 3.91)	<0.01	1.08 (0.82, 1.42)	0.60	2.26 (1.46, 3.49)	<0.01
HTN	1.23 (0.97, 1.55)	0.09	1.74 (1.15, 2.63)	<0.01	1.15 (0.90, 1.47)	0.26	1.49 (0.95, 2.31)	0.08
HF	2.24 (1.03, 4.86)	0.04	3.26 (1.29, 8.22)	0.01	1.96 (0.90, 4.28)	0.09	3.15 (1.22, 8.15)	0.02
CCD	1.13 (0.72, 1.78)	0.59	1.24 (0.65, 2.38)	0.51	1.03 (0.64, 1.63)	0.91	0.94 (0.46, 1.89)	0.86
Asthma	1.19 (0.65, 2.20)	0.57	1.73 (0.71, 4.21)	0.23	1.13 (0.60, 2.12)	0.71	2.20 (0.86, 5.59)	0.10
COPD	3.66 (1.57, 8.51)	<0.01	2.19 (0.60, 7.93)	0.23	3.19 (1.35, 7.52)	<0.01	1.39 (0.35, 5.59)	0.64
CKD	2.94 (1.37, 6.29)	<0.01	5.82 (2.23, 15.17)	<0.01	2.58 (1.19, 5.61)	0.01	5.35 (2.00, 14.31)	<0.01
MALIG	1.24 (0.70, 2.19)	0.46	2.81 (1.35, 5.88)	<0.01	1.17 (0.66, 2.06)	0.59	3.38 (1.59, 7.17)	<0.01
CLD	1.80 (0.92, 3.53)	0.08	1.48 (0.39, 5.63)	0.56	1.72 (0.87, 3.40)	0.12	1.26 (0.32, 5.02)	0.74
RDAD	1.67 (0.66, 4.24)	0.28	1.40 (0.17, 11.62)	0.75	1.63 (0.64, 4.17)	0.31	1.55 (0.18, 13.15)	0.69
DEMEN	0.82 (0.49, 1.37)	0.45	2.26 (1.29, 3.96)	<0.01	0.84 (0.50, 1.42)	0.52	2.62 (1.45, 4.72)	<0.01

Values are presented as estimate (95% confidence interval). BMI, body mass index; DM, diabetes mellitus; HTN, hypertension; HF, heart failure; CCD, cardiac conduction disease; COPD, chronic obstructive pulmonary disease; CKD, chronic kidney disease; MALIG, malignancy; CLD, chronic liver disease; RDAD, rheumatic disease/autoimmune disorder; DEMEN, dementia.

1 Model 1 is adjusted for age, sex, and BMI only; Model 2 is additionally adjusted for all of the examined comorbidities.
